# Low density lipoprotein receptor-related protein 1 expression correlates with cholesteryl ester accumulation in the myocardium of ischemic cardiomyopathy patients

**DOI:** 10.1186/1479-5876-10-160

**Published:** 2012-08-08

**Authors:** Roi Cal, Oriol Juan-Babot, Vicenç Brossa, Santiago Roura, Carolina Gálvez-Montón, Manolo Portoles, Miguel Rivera, Juan Cinca, Lina Badimon, Vicenta Llorente-Cortés

**Affiliations:** 1 Cardiovascular Research Center, CSIC-ICCC, Hospital de la Santa Creu I Sant Pau, Sant Antoni Ma Claret, 167, 08025, Barcelona, Spain; 2 Cardiology Service, IIB-Sant Pau. Hospital de la Santa Creu i Sant Pau, Universitat Autònoma de Barcelona, Barcelona, Spain; 3 ICREC Research Program, Fundació Institut d’Investigació en Ciències de la Salut Germans Trias i Pujol (IGTP), Badalona, Spain; 4 Research Center, Hospital Universitario La Fe, Valencia, Spain; 5 CIBER OBN, Instituto de Salud Carlos III, Cordoba, Spain

**Keywords:** Ischemic cardiomyopathy, LRP1, VLDLR, HIF-1α myocardial lipid accumulation

## Abstract

Our hypothesis was that overexpression of certain lipoprotein receptors might be related to lipid accumulation in the human ischemic myocardium. Intramyocardial lipid overload contributes to contractile dysfunction and arrhythmias in cardiomyopathy. Thus, the purpose of this study was to assess the effect of hypercholesterolemic LDL and hypertrigliceridemic VLDL dose on LRP1 expression in cardiomyocytes, as well as the potential correlation between LRP1 expression and neutral lipid accumulation in the left ventricle tissue from ischemic cardiomyopathy patients. Cell culture experiments include control and LRP1-deficient cardiomyocytes exposed to lipoproteins under normoxic and hypoxic conditions. Explanted hearts from 18 ICM patients and eight non-diseased hearts (CNT) were included. Low density lipoprotein receptor-related protein 1 (LRP1), very low density lipoprotein receptor (VLDLR) and low density lipoprotein receptor (LDLR) expression was analyzed by real time PCR and Western blotting. Cholesteryl ester (CE), triglyceride (TG) and free cholesterol (FC) content was assess by thin layer chromatography following lipid extraction. Western blotting experiments showed that protein levels of LRP1, VLDLR and HIF-1α were significantly upregulated in ischemic hearts. Immunohistochemistry and confocal microscopy analysis showed that LRP1 and HIF-1α were upregulated in cardiomyocytes of ICM patients. *In vitro* studies showed that VLDL, LDL and hypoxia exerted an upregulatory effect on LRP1 expression and that LRP1 played a major role in cholesteryl ester accumulation from lipoproteins in cardiomyocytes. Myocardial CE accumulation strongly correlated with LRP1 levels in ischemic hearts. Taken together, our results suggest that LRP1 upregulation is key for myocardial cholesterol ester accumulation in ischemic human hearts and that LRP1 may be a target to prevent the deleterious effects of myocardial cholesterol accumulation in ischemic cardiomyopathy.

## Introduction

The ischemic condition caused by decreased coronary flow is one of the most important factors leading to heart failure. Under ischemic conditions the myocardium undergos lipid accumulation in animal models [1-3] and humans [4]. In the ischemic heart, lipid vacuoles have been located in the periphery of the risk area [5,6]. While some authors have proposed a cardioprotective role for cytosolic lipids in the cardiomyocyte [7,8], others associate this phenomena to lipotoxicity [2,9]. In fact, it has been consistently reported that intramyocardial lipid overload significantly contributes to contractile dysfunction [10] and arrhythmias [11]. Recent work from our group demonstrated that myocytes exposed to high very low density lipoprotein (VLDL) doses depicts intracellular accumulation of neutral lipids, downregulation of SERCA2 expression, reduction of calcium transient amplitude, and SR calcium loading. These effects were exacerbated by submitting the cultured myocytes to an hypoxic environment [12]. Remarkably, a high prevalence of fat deposition has been found in areas of chronic myocardial infarction in humans [4]. The patients with fat deposition had larger infarctions, decreased wall thickening and impaired endocardial wall motion [4].

It is known from several years ago that triglyceride (TG)-rich lipoproteins (chylomicrons and VLDL) supply both triglycerides and cholesteryl esters (CE) to the heart [13-15]. At present, the lipoprotein receptors involved in TG and/or cholesterol uptake by the cardiomyocyte remain largely unknown. Indeed, a basal expression of lipoprotein receptors is essential to guarantee a correct energetic supply for the heart since its inhibition causes a marked decline in cardial mechanical function [15]. However, the upregulation of several lipoprotein receptors, including very low density lipoprotein receptor (VLDLR) [16] or low density lipoprotein receptor related protein 1 (LRP1) [17], has been previously reported to induce neutral lipid accumulation in cardiomyocytes under ischemic conditions. Moreover, both VLDLR [16] and LRP1 [17,18] share a HIF-1α-dependent mechanism of transcriptional activation by hypoxia in cultured cells. Our group has also shown that LRP1 expression and cholesteryl esters levels are found upregulated in the ischemic myocardium of an *in vivo* porcine model of acute myocardial infarction [17]. LRP1 is also upregulated by extracellular matrix-aggregated LDL in cultured human vascular smooth muscle cells and by hypercholesterolemia in the porcine vascular wall [19].

According to our previous work in this field, we hypothesized that LRP1 may be upregulated by lipids in cardiomyocytes playing a key role in the ischemia-induced neutral lipid accumulation in human heart. Thus, the purpose of this study was to assess the effect of hypercholesterolemic LDL and hypertrigliceridemic VLDL dose on LRP1 expression in cardiomyocytes, as well as the potential correlation between LRP1 expression and neutral lipid accumulation in the left ventricle tissue from patients with ischemic cardiomyopathy.

## Material and methods

### Collection of human samples

A total of 18 explanted human hearts from ischemic cardiomiopathy patients was collected and immediately processed. In particular, these patients were undergoing cardiac transplantation at Sant Pau Hospital, Barcelona and La Fe Hospital, Valencia. Clinical data, electrocardiogram, Doppler echocardiography, hemodynamic studies, and coronary angiography were available on all patients. All patients were functionally classified according to the New York Heart Association (NYHA) criteria, and were receiving medical treatment according to the guidelines of the European Society of Cardiology [20], with diuretics 90 %, angiotensin-converting enzyme inhibitors 87 %, β-blockers 50 %, aldosterone antagonists 70 %, digoxin 49 % and statins 80 %.

Eight non-diseased hearts were also obtained from donors with neurological death caused by traffic accident. The hearts were initially considered for cardiac transplantation but were subsequently deemed unsuitable for transplantation either because of blood type or size incompatibility. All donors had normal LV function and no history of myocardial disease or active infection.

Transmural samples were taken from the infarct border zone, and were immediately stored at −80 °C. The project was approved by the local Ethics Committee (Biomedical Ethics Committee of “La Fe, Valencia” and “Sant Pau, Barcelona”, Spain) and conducted in accordance with the guidelines of the Declaration of Helsinki. All patients gave written informed consent that was obtained according to our institutional guidelines.

### Tissue homogenization

Frozen ventricular tissues (25 mg) were pulverized using a mortar and a pestle in liquid nitrogen. Sample were then homogenized in TriPure isolation reagent (Roche Molecular Biochemicals) for total RNA and protein extraction according to manufacturer’s instructions.

### HL-1 cardiomyocyte cell culture

The murine HL-1 cell line was generated by Dr. W.C. Claycomb (Louisiana State University Medical Centre, New Orleans, Louisiana, USA) and kindly provided by Dr. U Rauch (Charité-Universitätmedizin Berlin). These cells show cardiac characteristics similar to those of adult cardiomyocytes. LRP1-deficient cardiomyocytes were generated as previously described [17]. Control and LRP1-deficient HL-1 cardiomyocytes were maintained in Claycomb Medium (JRH Biosciences, Lenexa, KS, USA) supplemented with 10 % fetal bovine serum (FBS) (Invitrogen Corporation, Carlsbad, CA, USA), 100 μM norepinephrine, 100 units/mL penicillin, 100 μg/mL streptomycin, and L-Glutamine 2 mM (Sigma Chemical Company, St. Louis, MO, USA) in plastic dishes, coated with 12.5 μg/mL fibronectin and 0.02 % gelatin, in a 5 % CO_2_ atmosphere at 37 °C.

### VLDL and LDL preparation

Human VLDL (d_1.019_-d_1.019_ g/mL) and LDL (d_1.009_-d_1.063_ g/mL) were obtained from pooled sera of normocholesterolemic volunteers. VLDL and LDL preparations were less than 24 hours old and without detectable levels of endotoxin (Limulus Amebocyte Lysate test, Bio Whittaker). Aggregated LDL (agLDL) was prepared by vortexing LDL in PBS at room temperature. The formation of LDL aggregates was performed as previously described [21-23]. The ultrastructure of agLDL obtained by vortexing was similar to that of LDL modified by versican [22], one of the main chondroitin sulfate proteoglycans structuring the arterial intima.

### Exposure of cardiomyocytes to VLDL and LDL under normoxic and hypoxic conditions

Cells were exposed to normoxia (21 % O_2_) in a Nirco gas incubator with gas mixtures consisting of 74 % N_2_ and 5 % CO_2_ or to hypoxia (1 % O_2_) in a Hypoxic/Anoxic Workstation: H35 (Don Whitley Scientific Ltd.) with 94 % N_2_ and 5 % CO_2_. Lipoproteins were added and maintained for the last 12 hours of exposure to normoxic or hypoxic conditions (24 hours). Cells were then harvested in TriPure Reagent (Roche) for PCR and Western blot analysis or in NaOH 0.1 M for lipid extraction and thin layer chromatography**.**

### RNA extraction and cDNA synthesis

Total RNA was extracted from fresh frozen tissue or cultured HL-1 cardiomyocytes using TriPure isolation reagent (Roche Molecular Biochemicals) and the RNeasy mini kit (Qiagen, Hilden, Germany) according to manufacturer’s instructions. Extracted RNA was eluded in 25 μL of nucleases-free water. RNA yield and quality were assessed by agarose electrophoresis and spectrophotometry, and then stored at −80°C until was used. RNA was digested with DNase I (Invitrogen). One μg of total RNA was used for cDNA synthesis according to the protocol provided with the HighCapacity cDNA Reverse Transcription kit (Applied Biosystems, Foster City, CA, USA). Recombinant RNasin Ribonuclease Inhibitor (Applied Biosystems) was added to prevent RNase-mediated degradation. The cDNA was also stored at –20 C.

### Gene expression analyses by RT-PCR

Gene expression analyses of *LRP1VLDLR*, and *LDLR* mRNA were performed at mRNA level by quantitative real-time reverse transcriptase-polymerase chain reaction (q-RT-PCR). Specific primer and fluorescent TaqMan probe for *LRP1VLDLR* and *LDLR* were selected within a list of predesigned assays (Assays-on-Demand LRP1 (Hs00233999_m1), VLDLR (Hs01045922_m1) and LDLR (Hs00181192_m1) (Applied Biosystems). *18srRNA* (4319413E) was used as a housekeeping gene. We mixed 5 μl of single-stranded cDNA (equivalent to 100 ng of total RNA) with 1 μl of 20x TaqMan Gene Expression Assays for each Assay-on-Demand, 10 μl of TaqMan Universal PCR Master Mix, and 4 μl of nucleases-free water. After gentle mixing, the mixture was transferred into a real-time PCR microplate. The Real-time PCR microplate was sealed, centrifuged, and then was placed in the sample block of an Applied Biosystems 7300 Real Time PCR System (Applied Biosystems). The thermal cycling conditions were 2 min at 50°C and 10 min at 95°C, followed by 40 cycles of 15 s at 95°C and 1 min at 60°C. Expression levels were measured in triplicate. The threshold cycle (Ct) values were normalized to the housekeeping gene [17,18].

### Western blotting

Total protein was extracted from fresh frozen tissue or HL-1 cell cultures using TriPure isolation reagent (Roche Molecular Biochemicals). Proteins were analyzed by Western blot analysis as previously described [17,18]. Blots were incubated with monoclonal antibodies against human LRP1 (β*-*chain, clone 8B8 RDI 61067), VLDLR (Santa Cruz Biotechnology, Inc, D-17, sc-11823), HIF-1α (Santa Cruz Biotechnology, H-206, sc-10790), LDLR (Epitomics, EP1553Y, 1956–1), VEGF (Santa Cruz Biotechnology, Inc, A2611, sc-152) and mouse monoclonal anti-Troponin T (Thermo scientific MS-295). Equal protein loading in each lane was verified staining filters with Pounceau and also by incubating blots with monoclonal antibodies against β-actin (Abcam, ab8226).

### Lipid extraction and semi-quantitative analysis of cholesteryl ester, free cholesterol and triglyceride content of cardiomyocytes and myocardium

HL-1 cardiomyocytes were exhaustively washed and harvested in NaOH 0.1 M following the lipoprotein incubation period. In the animal experimental model, one portion of myocardial tissue (5 mg) was also homogenized in NaOH 0.1 M. Lipids were extracted as previously described [17,18] and CE, FC and TG content was analyzed by thin layer chromatography.

The organic solvent was removed under an N_2_ stream, the lipid extract redissolved in dichloromethometane and one aliquot was partitioned by thin layer chromatography (TLC). TLC was performed on silica G-24 plates. The different concentrations of standards (a mixture of cholesterol, cholesterol palmitate, triglycerides, diglycerides and monoglycerides) were applied to each plate. The chromatographic developing solution was heptane/diethylether/acetic acid (74:21:4, vol/vol/vol). The spots corresponding to cholesteryl esters (CE), triglycerides (TG) and free cholesterol (FC) were quantified by densitometry against the standard curve of cholesterol palmitate, triglycerides and cholesterol, respectively, using a computing densitometer.

### Immunohistochemical analysis

Hearts were obtained from human transplant operations. Immediately after surgical excision, myocardium was cut in appropriated blocks. Myocardial tissues were immersed in fixative solution (4 % paraformaldehyde), embedded in paraffin, cut into 5 μm thick serial sections and placed on poly-L-lysine coated slides. The primary antibodies were rabbit monoclonal anti-LRP1 (Epitomics 2703 dilution 1:100), mouse monoclonal anti-Troponin T (Thermo scientific MS-295, dilution 1:100) and mouse monoclonal anti-HIF-1α (Novus NB100-105, dilution 1:50). Antigen retrieval was required before performing immunohistochemical staining of Troponin T and HIF-1α. In a set of experiments, before incubation with primary antibody (2 hours), sections were washed and endogenous peroxidase activity suppressed with H_2_O_2_. Non-specific binding was blocked with an appropiate serum. The primary antibodies were detected using the avidin-biotin immunoperoxidase technique. The sections were incubated with an appropriate biotinylated secondary antibody (1:200, Vector®). 3,3’-diaminobenzidine-haematoxylin chromogen was used for nuclear stain. The images were captured by Nikon Eclipse 80i microscope and digitized by Retiga 1300i Fast camera. Magnification (240X).

In other set of experiments, cryosections were subsequently incubated with a Cy3-conjugated secondary antibody (Jackson Immuno Research Laboratories) at 37 °C for 1 h. Slices were finally counterstained for 10 min with Hoechst 33342 (Sigma), and analyzed under a TCS SP5 confocal microscope (Leica).

### Statistical analysis

Results are expressed as mean ± standard deviation (SD). Statistical significance between groups was analyzed by one-way analysis of variance (ANOVA) followed by a *post-hoc Tamhane* test. Correlation analysis was performed according to Pearson. Statistics were calculated using Statistical software package Statview (SPSS) for Windows. A value of *P* < 0.05 was considered significant.

## Results

### Clinical characteristics of patients

The clinical and echocardiographic characteristics of patients are summarized in Table 1. These patients were all symptomatic, had a NYHA functional classification of III-IV and were previously diagnosed with significant comorbidities including hypertension, hypercholesterolemia, obesity, and diabetes mellitus. Patients (93 %) were mostly men with a mean age of 54.53±2.67 years and had at least one vessel affected by atherosclerosis. Left ventricular end-diastolic diameter was 67.00 ± 2.45. Eight non-diseased donor hearts were used as control (CNT) (60 % male, mean age 55±3 years, and EF > 50 %). A high percentage of patients (67 %) were subjected to reperfusion techniques, also indicative of peri-infarct ischemia.

**Table 1:** Clinical and echocardiographic characteristics from whom explanted ischemic hearts were obtained

	**ICM (n = 18)**
Age (years)	54.53 ± 2.67
Gender male (%)	93 %
Prior hypertension (%)	46
Diabetes mellitus (%)	27
Obesity (%)	20
Total cholesterol (nmol/L)	3.90 ± 0.28
Perfusion abnormalities* (%)	67 %
Ejection fraction (%)	25.86 ± 1.80
Intraventricular septum in diastole (mm)	11.08 ± 0.47
Left ventricular posterior wall in diastole (mm)	9.67 ± 0.62
Left ventricular end-diastolic diameter (mm)	67.00 ± 2.45
Left ventricular end-systolic diameter (mm)	58.50 ± 3.94
Diuretics	90
Angiotensin-converting enzymes inhibitors	87
β-blockers	50
Aldosterone antagonist	70
Digoxin	49
Statins	80

ICM, ischemic cardiomyopahhy; *the patients with perfusion abnormalities were considered those subjected to coronary interventions (i.e. by-pass, angioplasty, stents and others).

### Myocardial lipoprotein receptor expression

**Figure 1:**
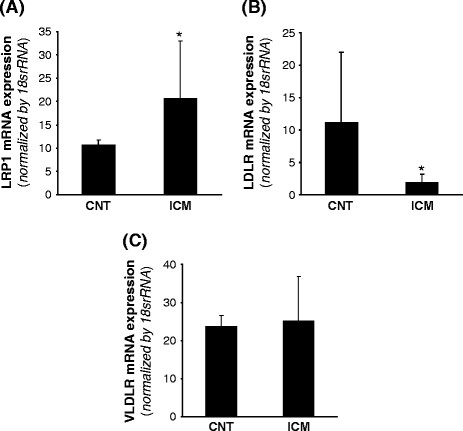
**LRP1, VLDLR and LDLR mRNA expression in control and ICM hearts. **Frozen myocardial tissue (5 mg) was homogenized in Tripure reagent and RNA isolated as explained in Methods. LRP1 (**A**), LDL receptor (**B**) and VLDLR (**C**) mRNA expression levels were analyzed by real time PCR. Data were processed with a specially designed software program based on Ct values of each sample and normalized to 18srRNA as endogenous control. *P  < 0.05 *versus *CNT. CNT, controls; ICM, ischemic cardiomyopathy patients.

**Figure 2:**
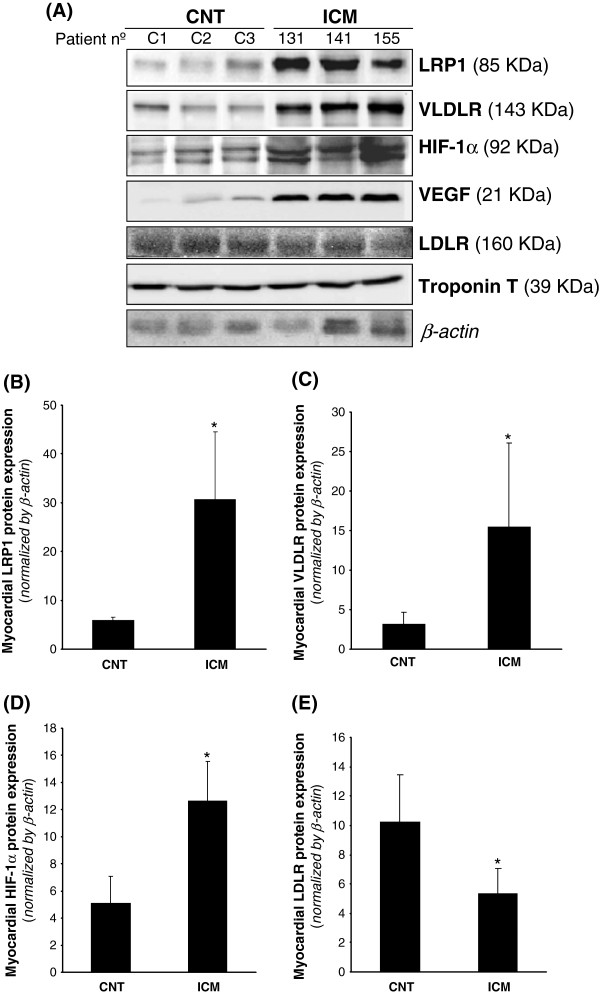
**LRP1, VLDLR and LDLR protein levels in control and ICM hearts. **Representative Western blot analysis (**A**) showing LRP1, VLDLR, HIF-1α, VEGF, LDLR and troponin T protein expression in three controls and three ICM patients. Bar graphs showing the mean ± SD of protein LRP1 (**B**), VLDLR (**C**), HIF-1α (**D**) and LDLR (**E**) band quantification. Unchanged levels of β-actin were shown as loading control and used to normalize protein bands. *P  < 0.05 *versus *CNT. CNT, controls; ICM, ischemic cardiomyopathy patients.

LRP1 expression was analyzed at mRNA level by real time PCR and at the protein level by Western blot analysis from the same sample. As shown in Figure 1A, LRP1 mRNA expression was significantly higher in ICM than in control group (ICM: 20.57±12.41 *vs* CNT: 10.66±1.13, *P* < 0.05). In contrast, LDLR mRNA expression (Figure 1B) was significantly lower in ICM compared to controls (ICM: 1.89±1.29 vs CNT: 11.14±10.90, *P* < 0.05). No differences were found in VLDLR mRNA expression between controls and ICM hearts (Figure 1C). In agreement with real-time PCR data, Western blot analysis showed that LRP1 protein expression was significantly higher in ICM than in CNT hearts (ICM: 30.62 ± 13.93 *vs* CNT: 5.92 ± 0.66, *P* < 0.05) (Figure 2A & B). Additionnally, VLDLR protein expression was significantly upregulated in ICM compared to CNT samples (ICM: 15.48 ± 10.62 *vs* CNT: 3.68 ± 2.98, *P* < 0.05) (Figure 2A & C). In order to know whether HIF-1α transcription factor may play a role on lipoprotein receptor upregulation in ICM samples, we analyzed HIF-1α protein levels by Western blotting and used VEGF as a positive control. HIF-1α protein levels were significantly higher in ICM in comparison with CNT hearts (ICM: 12.63 ± 2.92 vs CNT: 5.11 ± 1.97, *P* < 0.05) (Figure 2A & D), as well as VEGF significantly increased in ICM samples. In contrast to LRP1 and VLDLR, LDLR protein expression was significantly lower in ICM samples (Figure 2A & E).

**Figure 3:**
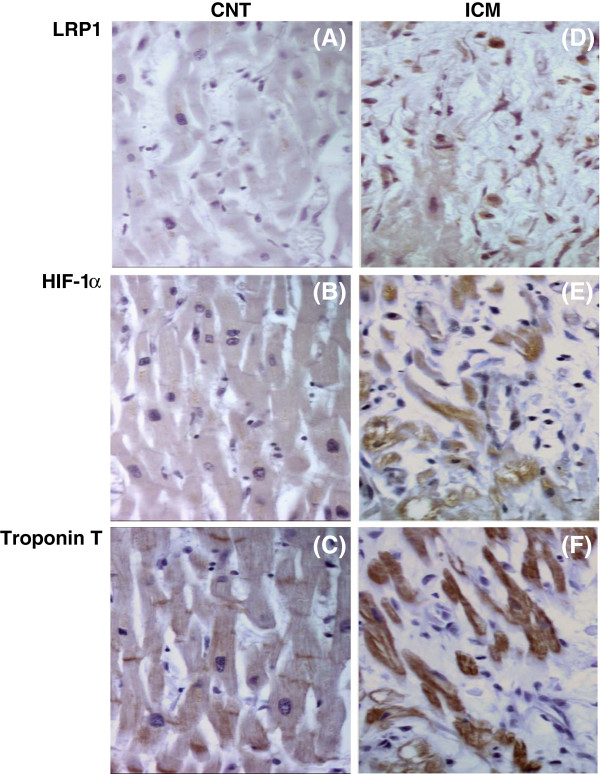
**LRP1, HIF-1**α **and troponin T staining in control and ICM hearts. **Representative immunohistochemical analysis of LRP1 (panel **a** &**d**), HIF-1α (panel **b** &**e**) and troponin T (panel **c** &**f**) staining in consecutive slides of CNT and ICM samples. CNT, controls; ICM, ischemic cardiomyopathy. Magnification x 240.

**Figure 4:**
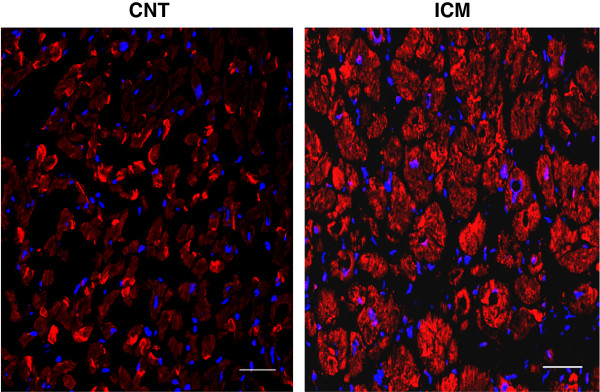
**Confocal microscopy analysis of LRP1 expression in control and ICM hearts. **Representative confocal microscope images showing specific LRP1 detection in myocardium from one ICM patient subjected to reperfusion and one from a control subject. Nuclei were counterstained with Hoechst 33342 (blue). Scale bars = 50 μm (magnification x 400).

Immunohistochemical analysis (Figure 3A) showed that LRP1 (**panel a**) and HIF-1α (**panel b**) staining were undetectable within control myocardium. On the contrary, there was a positive staining for LRP1 (**panel d**) and HIF-1α (**panel e**) in the myocardium of ICM patients. Confocal microscopy showed higher cardiomyocyte LRP1 staining in ICM patients compared to control subjects (Figure 4).

### Effect of VLDL and LDL on LRP1 protein expression in cardiomyocytes under normoxic and hypoxic conditions

**Figure 5:**
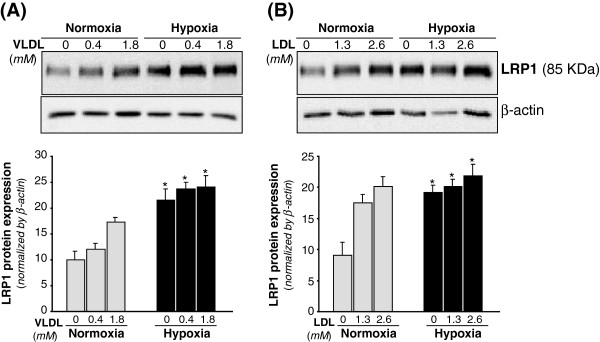
**Dose-dependent changes of LRP1 protein levels in cardiomyocytes exposed to VLDL or LDL under normoxic or hypoxic conditions. **HL-1 cardiomyocytes were submitted to normoxia or hypoxia for 24 hours either in absence or presence of increasing dose of VLDL (**A**) or LDL (**B**) for the last 12 hours. Representative Western blot analysis showing LRP1 bands. Unchanged levels of β-actin were shown as loading control and used to normalize LRP1 bands. Results were shown as mean ± SEM of three independent experiments performed in duplicate.*P  < 0.05 *versus *normoxic cells. # *P * < 0.05 *versus *cells incubated in absence of lipoproteins.

**Figure 6:**
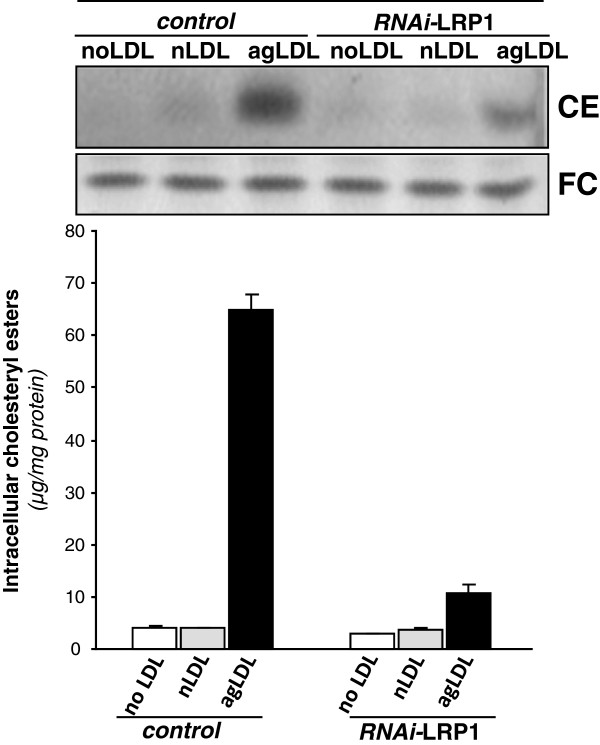
**Role of LRP1 in the uptake of modified lipids by cardiomyocytes. **Control and LRP1-deficient HL-1 cardiomyocytes were submitted to or hypoxia for 24 hours either in absence or presence of nLDL or agLDL (0.6 mM) for the last 12 hours. Cells were exhaustively washed and collected in NaOH 0.1 M as explained in Methods. Thin layer chromatography showing CE and FC bands and histograms with their quantification. Results are expressed as micrograms per milligram of protein and shown as mean ± SEM of three experiments performed in triplicate. ξ*P * < 0.05 *versus *control cells.

VLDL (1.8 mM) (Figure 5A) and LDL (2.6 mM) (Figure 5B) significantly increased LRP1 protein by 1.72-fold and 2-fold, respectively. Hypoxia *per se* also exerted a significant upregulatory effect on LRP1 expression in HL-1 cardiomyocytes in agreement with previous results [17]. Previous studies from our group showed that LRP1 play a key role for aggregated LDL (agLDL) uptake in human vascular smooth muscle cells [21-23]. To know whether LRP1 may also take modified lipids in cardiomyocytes, we exposed control and LRP1-deficient cardiomyocytes [17] to agLDL. As shown in Figure 6, LRP1 deficiency significantly decreased the strong intracellular CE accumulation (65 ± 3 μg CE/mg protein) derived from agLDL (0.6 mM) in HL-1 cardiomyocytes. In contrast, LRP1 deficiency did not influence the slight intracellular CE (10.6 ± 0.2 μg CE/mg protein) induced by native LDL (0.6 mM) in cardiomyocytes. 

### Myocardial neutral lipid content and correlation with lipoprotein receptor expression

**Figure 7:**
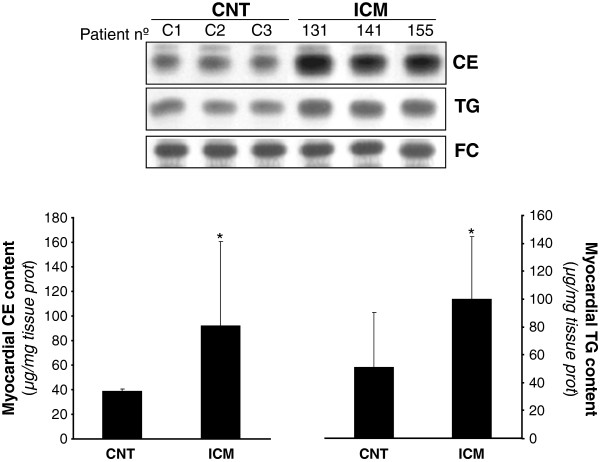
**Myocardial cholesteryl ester (CE), triglyceride (TG) and free cholesterol content (FC) in controls and ICM hearts.** Representative thin layer chromatography showing CE, TG and FC bands in three control and three ICM patients, and bar graphs showing the mean ± SD of myocardial colesteryl ester and triglyceride bands in CNT (n = 8) and ICM (n = 18) groups. CNT, controls; ICM, ischemic cardiomyopathy. Results are expressed as micrograms per mg of tissue.

Myocardial lipid content was analyzed by thin layer chromatography following lipid extraction. Figure 7 shows representative CE, TG and FC bands from ICM patients and controls. Both myocardial CE (ICM: 92.2±68.3 *vs* CNT: 39±18, *P* < 0.05) and TG (ICM: 100.2±44.4 *vs* CNT: 50.8±39.4*, P* = 0.04) content was significantly increased in ICM *vs* CNT. FC content of myocardium was unaltered by disease.

**Figure 8:**
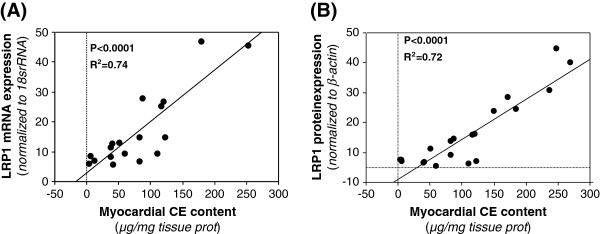
**Correlation between LRP1 and CE content in the myocardium of ischemic cardiomyopathy patients. **Analysis of correlation between myocardial cholesteryl ester content and LRP1 mRNA (**A**) or LRP1 protein (**B**) expression in ischemic cardiomyopathy patients (n = 18).

Myocardial CE strongly correlated with LRP1 mRNA (R^2^ = 0.74, *P* < 0.0001) (Figure 8A) and LRP1 protein expression (R^2^ = 0.72, *P* < 0.0001) (Figure 8B). Although to a minor extent, myocardial CE also correlated with VLDLR protein expression (R^2^ = 0.398, *P* < 0.012). In contrast, myocardial TG content did not show any correlation with LRP1 mRNA or protein expression.

## Discussion

Taken together, our results demonstrate for the first time that myocardial LRP1 expression and cholesteryl ester content appear strongly increased and interrelated in the myocardium of ischemic cardiomyopathy patients. In particular, we show that LRP1 is upregulated in ischemic human hearts at mRNA and protein levels, although to a different extent. This difference in the quantitative modulation of LRP1 at mRNA and protein levels by hypoxia has been previously shown in human VSMC and it may be explained at least in part by the long half-life time of LRP1 protein [18].

The upregulation of HIF-1α protein levels in ICM hearts suggests that in patients, as previously shown in cultured cardiomyocytes [17] and vascular cells [18], HIF-1α may participate in myocardial LRP1 upregulation in ischemic hearts. However, further studies are required to know the precise role of HIF-1α on myocardial LRP1 overexpression in ischemic cardiomyopathy. In the group of patients included in this study, there was a high percentage of those with documented perfusion abnormalities, indicative of peri-infart ischemia. Moreover, enrolled patients had at least one vessel affected by atherosclerosis. Therefore, cardiovascular risk factor such as hypercholesterolemia or hypertension likely modulate LRP1 expression in the myocardium, as previously reported by our group in the vascular wall [19,24-26]. Indeed, the presented results demonstrate that high VLDL and LDL dose strongly increased LRP1 protein levels in cardiomyocytes.

Ischemia has been reported to upregulate VLDLR expression in cultured cardiomyocytes [12,16] and mice [16]. We also found VLDLR protein overexpressed in a porcine model of acute myocardial infarction [17] and in human ischemic hearts. Although VLDLR has been reported to be upregulated at mRNA level in myocardial biopsies taken from patients scheduled for coronary bypass surgery [16], we did not observe alterations of mRNA VLDLR expression levels in explanted hearts from ICM patients. It has been previously suggested that cyclosporine, an immunosuppressant used for the treatment of transplant recipients, inhibits VLDLR mRNA expression in the myocardium of ischemic cardiomyopathy patients [16]. In contrast to LRP1 expression, LDLR mRNA expression was lower in ischemic hearts compared to controls in agreement with our previous results in hypoxic cardiomyocytes [12,17] and vascular cells [18], suggesting that the classical LDLR plays no role in the neutral lipid accumulation associated to ischemic myocardium.

A significant finding of this study is the close association between LRP1 expression and cholesteryl ester accumulation in ischemic human hearts. Although this study does not provide information about the mechanisms involved in the link between LRP1 expression and CE accumulation, previous studies in our group have shown that LRP1 plays an essential role in the selective VLDL-cholesteryl ester uptake by hypoxic cardiomyocytes [17]. Additionnally, results from the present study show that cardiomyocyte LRP1, as vascular LRP1 [21-23], internalizes cholesteryl esters from modified lipoproteins. Taken together, these results support that CE overaccumulation in human ischemic hearts may be caused by the capacity of LRP1, when overexpressed by hypoxia, to take up cholesterol from lipoproteins. Previously, it has been shown that increased expression of VLDLR is essential for the accumulation of triglycerides in hypoxic cardiomyocytes and ischemic myocardium in mice [16]. These authors also found a slight but significant correlation between VLDLR mRNA and ORO staining. Here, we found that VLDLR, although to a minor extent that LRP1 expression, also correlated with CE accumulation in ischemic heart. In contrast, we did not observe any significant correlation between LRP1 or VLDLR expression and myocardial TG content. This lack of correlation may be related to the multiple pathways that influence myocardial TG content, that may derive from endogenous synthesis [27,28], diffusion of albumin-associated fatty acids [29] or lipoprotein-TG-hydrolysis by LpL [30,31].

In addition, also in agreement with our previous studies *in vitro*[12,17] and *in vivo*[17], we evidenced that in the myocardium of ischemic cardiomyopathy patients there was not only triglyceride but also cholesteryl ester accumulation. This is an interesting observation since most of the previous studies assumed that myocardial neutral lipid accumulation consist only in triglycerides [1-3,16]. Indeed, a precise knowledge about the mechanism of myocardial lipid uptake is required to prevent the deleterious consequences of this process in cardiac functionality.

### Limitations and considerations of the study

The complexity of cardiomyopathy and heart failure originated by myocardial infarction cannot be afforded by testing the effect of hypoxia (1 % O_2_) in cultured cardiomyocytes or the effect of coronary occlusion on porcine myocardial gene expression. However, these *in vitro* and *in vivo* models have been useful to identify new genes involved in pathophysiological mechanisms underlying the deleterious effect of hypoxia on cardiac function. Recently, our group has previously identified LRP1 as key receptor for cholesteryl ester accumulation in hypoxic cardiomyocytes and ischemic porcine myocardium [17]. Results from the present study show that there is a strong LRP1 upregulation that significantly correlates with cholesteryl ester accumulation in ischemic cardiomyopathy patients. Therefore, our results suggest that cardiac alterations associated to the deleterious effects of myocardial cholesterol accumulation may be modulated through LRP1 targeting in heart failure and cardiomyopathy.

The clinical consequences of lipid accumulation under ischemia have not been directly addressed in this study but it is known that there is a close association between CE content of sarcoplasmatic reticulum and SERCA-2 suppression [32]. Additionally, hypercholesterolemia exacerbates myocardial necrosis and apoptosis in the setting of ischemia-reperfusion [33]. It is also known that hypoxia exerted a downregulatory effect on SERCA2 expression through HIF-1α [34]. Our group demonstrated that hypoxia exacerbates the alterations in calcium handling induced by VLDL through potentiation of SERCA2 downregulation [12], and that the percentage of waves was reduced in calcium-overloaded-LRP1-deficient cardiomyocytes under stimulation. Taken together, our results consistently point out that LRP1 is a potential molecular target to prevent myocardial CE accumulation, and thus the cardiac alterations induced by cholesterol loading in ischemic cardiomyopathy.

## Competing interests

The authors declare that they have no competing interests.

## Authors’ contributions

RC performed *in vitro* experiments and molecular analysis, OJB designed and set up immunohistochemical analysis, SR and CGM performed confocal microscopy experiments, OJB, VB, MP and JMR collected the data from the patients. JC and LB oversaw the manuscript. VLLC conceived the study, designed and prepared the manuscript. All authors read and approved the final manuscript.
